# Hari-Kari

**Published:** 1904-07

**Authors:** 


### HARI-KARI.

THE name Hari-Kari, or euphemistically happy despatch, is that given to the popular method of suicide among the Japanese. It was said to be performed in this manner: The abdomen was cut from side to side and then from above downward—a crucial incision. It would appear that this is incorrect as death would not be immediate in this form of injury nor would there be many with sufficient fortitude to complete the cross incision after the first was made. It is more likely that a knife is introduced into the abdomen, the edge being then swept across the front of the spinal column severing the ascending vena cava and the abdominal aorta. With such a wound death would occur almost immediately.

The Japanese resort to the happy despatch from motives that appear to our minds to be absolutely trivial.

